# A framework incorporating the impact of exposure scenarios and application conditions on risk assessment of chemicals applied to skin

**DOI:** 10.1186/2193-9616-1-10

**Published:** 2013-06-14

**Authors:** Yuri Dancik, John A Troutman, Joanna Jaworska

**Affiliations:** The Procter & Gamble Company, Temselaan 100, Strombeek-Bever, 1853 Belgium; The Procter & Gamble Company, Cincinnati, OH 45253 USA

**Keywords:** Forward and reverse dosimetry, Dermal exposure, Skin penetration kinetics, PBPK

## Abstract

**Purpose:**

1. To develop a framework for exposure calculation via the dermal route to meet the needs of 21st century toxicity testing and refine current approaches; 2. To demonstrate the impact of exposure scenario and application conditions on the plasma concentration following dermal exposure.

**Method:**

A workflow connecting a dynamic skin penetration model with a generic whole-body physiologically-based pharmacokinetic (PBPK) model was developed. The impact of modifying exposure scenarios and application conditions on the simulated steady-state plasma concentration and exposure conversion factor was investigated for 9 chemicals tested previously in dermal animal studies which did not consider kinetics in their experimental designs.

**Results:**

By simulating the animal study scenarios and exposure conditions, we showed that 7 studies were conducted with finite dose exposures, 1 with both finite and infinite dose exposures (in these 8 studies, an increase in the animal dose resulted in an increase in the simulated steady-state plasma concentrations (*C*_p,ss_)), while 1 study was conducted with infinite dose exposures only (an increase in the animal dose resulted in identical *C*_p,ss_). Steady-state plasma concentrations were up to 30-fold higher following an infinite dose scenario vs. a finite dose scenario, and up to 40-fold higher with occlusion vs. without. Depending on the chemical, the presence of water as a vehicle increased or decreased the steady-state plasma concentration, the largest difference being a factor of 16.

**Conclusions:**

The workflow linking Kasting’s model of skin penetration and whole-body PBPK enables estimation of plasma concentrations for various applied doses, exposure scenarios and application conditions. Consequently, it provides a quantitative, mechanistic tool to refine dermal exposure calculations methodology for further use in risk assessment.

## Background

Traditional toxicology heavily relies on animal experimentation to assess the risk of human exposure to chemicals. Concerns with this method include overuse of animals, expense, low throughput and limited relevance to human toxicity (Bhattacharya et al. 2011). To address these issues, the U.S. National Research Council (NRC) published *Toxicity Testing in the 21*st *Century: A Vision and a Strategy* in 2007 (NRC 2007). This report details the need to base toxicity assessment on the response of toxicity pathways in *in vitro* assays in combination with dose–response and extrapolation modeling (Bhattacharya et al. 2011).

Within the context of 21st century toxicology, there is a need to develop approaches that allow risk assessment to be based on an internal dose metric (plasma/blood or a target organ) rather than the external applied dose (Thompson et al. 2008; Coecke et al. 2012; Gundert-Remy et al. 2013). The internal dose metric allows to better establish a dose–response relationship and to reduce uncertainties inherent to traditional risk assessments (Thompson et al. 2008; Boekelheide and Andersen 2010). Internal dose calculations from the external dose, so called forward dosimetry, are plentiful in the literature and are done using PBPK models (Clewell et al. 2008). A majority of the forward dosimetry work deals with oral dosing because most of the *in vivo* animal testing has been done using the oral route of exposure.

To extrapolate *in vitro* data, the 21st century toxicity testing paradigm requires, in addition to forward dosimetry, calculations for estimating an external dose corresponding to an internal concentration. This process is referred to as reverse dosimetry (Clewell et al. 2008). When population variability is not considered, reverse dosimetry amounts to the simple calculation of an exposure conversion factor (ECF) linking the internal plasma concentration to the external exposure dose. Recently, this approach was used to calculate a daily human oral dose needed to produce the *in vivo* steady-state blood concentration of chemicals equivalent to an *in vitro* AC_50_ (concentration at which activity is 50% of its maximum) or a LEC (lowest effective concentration) (Rotroff et al. 2010; Judson et al. 2011; Wetmore et al. 2012).

In some contexts, the dermal route of exposure is of equal if not greater importance to the oral route. Skin is the most important site of exposure for consumer products, pesticides and solvents (Buist et al. 2009; Ngo et al. 2009). It is also significant for industrial solvents, which, despite their volatility, can penetrate the skin due to high lipophilicity (Semple 2004; Weschler and Nazaroff 2012). There is therefore the need to further develop both forward and reverse dosimetry approaches applicable to the dermal route, relevant to human skin and applicable to a variety of realistic exposure conditions. To achieve this, one needs to use a transient model of skin penetration. Till now such models were not available because of the focus on modeling in the steady-state driven by the availability of experimental data.

Taking advantage of the progress made in transdermal transport modeling and in particular the availability of the *in vivo* human physiologically-based skin penetration model developed by Kasting and co-workers (described in (Dancik et al. 2013)), we were able to develop a workflow for forward and reverse dosimetry for the dermal route. To this end, Kasting’s *in vivo* skin penetration model was connected with a generic whole-body PBPK model to calculate plasma concentration. Next, we investigated the impact of varying exposure scenarios and application conditions on plasma concentrations. The choice of the exposure scenarios in this study stems from the exposure conditions used in the *in vivo* dermal studies we investigated. While the influence of exposure scenarios and application conditions has been addressed and modelled conceptually by others (Krüse and Verberk 2008; Ngo et al. 2009), here we quantitatively show the impact of these parameters on the plasma concentrations. This allows us to further build support for using internal exposure as a metric in modern risk assessment.

## Methods

### Selection of chemicals

Our work is part of the ChemScreen project, the goal of which is the development of animal-free screening methods for reproductive toxicants (ChemScreen 2010). For this reason we selected 9 chemicals whose reproductive and/or developmental toxicity following dermal exposure has been studied in animals (Table 1).The chemicals are Bayrepel (BR), Capsaicin (CAP), Diethylene glycol monomethyl ether (DGMME), Diethylene glycol mono-n-butyl ether (DGMBE), Dimethylformamide (DMF), 2-Ethylhexanol (2-EH), 2-Methoxypropyl-1-acetate (MPA), 2-Methoxyethanol (2-ME) and Thioglycolic acid (TGA). Within the ChemScreen project, work is currently underway to establish the concentrations yielding toxicity in *in vitro* assays for these chemicals. We will ultimately relate these *in vitro* concentrations to external exposure doses.

**Table 1:** **Overview of chemicals, main physicochemical input parameters and** ***in vivo*** **animal studies**

Chemical	Structure	Physicochemical properties					***In vivo*** animal studies		
		***MW*** [g/mol]	log ***K***_o/w_^(a)^	***P***_vapor_ [mmHg] ^(b)^			Applied doses ^(c)^ [mg/kg/day]	Exposure scenario and application conditions	Reference
				measured at 25°C	predicted at 25°C	predicted at 32°C			
BR		229.20	1.80	Not available in EpiWin	8.3 · 10^-6^	2.3 · 10^-5^	50, 200, **400**	20-day exposure, no removal undiluted, unoccluded	(Astroff et al. 2000)
CAP		305.20	3.20	Not available in EpiWin	1.3 · 10^-8^	4.3 · 10^-8^	**64**, **96**, 128 ^(d)^	11-day exposure, daily removal after at least 3 h ^(e)^ , undiluted ^(f)^, occluded	(Chanda et al. 2006)
DGMME		120.08	−1.16	2.5 · 10^-1^	1.1 · 10^-1^	2.1 · 10^-1^	**50**, 250, 750	13-day exposure, no removal, undiluted, occluded	(Scortichini et al. 1986)
DGMBE		162.13	0.44	2.2 · 10^-2^	1.1 · 10^-2^	2.2 · 10^-2^	100, 300, **1000**	13-day exposure, daily removal after 4 h, 3 mL/kg water vehicle ^(g)^	(Nolen et al. 1985)
DMF		73.05	−0.83	3.9	3.5	5.4	100, **200**, 400	13-day exposure, daily removal after 6 h, undiluted, semi-occluded ^(h)^	(Hellwig et al. 1991)
2-EH		130.14	2.72	1.4 · 10^-1^	1.9 · 10^-1^	3.4 · 10^-1^	252, **840**, **2520** ^(i)^	10-day exposure, daily removal after 6 h, undiluted, occluded	(Tyl et al. 1992)
MPA		132.08	0.48	Not available in EpiWin	7.7	5.0	1000, **2000**	13-day exposure, daily removal after 6 h, undiluted, semi-occluded ^(h)^	(Merkle et al. 1987)
2-ME		76.05	−0.70	9.5	5.6	9.0	840	10-day exposure, daily removal after 6 h, undiluted, occluded	(Tyl et al. 1992)
TGA		91.99	0.14	8.7 · 10^-2^	4.2 · 10^-1^	6.9 · 10^-1^	10, 20, 25, **65** ^ (j)^	24-day exposure, daily removal after 6 h, 1:1 (v/v) ethanol (95%) – water vehicle, unoccluded	(Tyl et al. 2003)

*BR* Bayrepel, *CAP* Capsaicin, *DGMME* Diethylene glycol monomethyl ether, *DGMBE* Diethylene glycol mono-n-butyl ether, *DMF* Dimethylformamide, *2-EH* 2-Ethylhexanol, *MPA* 2-Methoxypropyl-1-acetate, *2-ME* 2-Methoxyethanol, *TGA* Thioglycolic acid. ^(a)^ ACDLabs ^(b)^ Values from EpiSuite. The final vapor pressure at the skin temperature, 32°C, is calculated from the EpiWin measured and predicted values as described in (Dancik et al. 2013). In case a measured value at 25°C is not available, only the predicted value at 32°C is used. ^(c)^ NOAELs or NOELs indicated in bold. ^(d)^ Values estimated from reported doses of 16, 24 and 32 mg/rat, assuming an average body weight of 250 g. 64 mg/kg/day is the maternal NOEL, 96 mg/kg/day is the developmental NOEL. ^(e)^ Taken as removal 3 h after application. ^(f)^ Dissolution of CAP in diethylene glycol monoethyl ether in the animal study was not taken into account in the skin penetration simulations. ^(g)^ Assumed non-occluded. ^(h)^ Assumed equivalent to occluded. ^(i)^ 840 mg/kg/day is NOAEL for maternal systemic toxicity; 2520 mg/kg/day is NOAEL for developmental toxicity. ^(j)^ The 10, 25 and 65 mg/kg/day doses were simulated.

### Calculation of dermal human doses from animal doses

In order to predict the penetration of the 9 chemicals in human skin, we scaled the dermal doses applied in the animal studies to human dermal doses. Permeant and vehicle doses applied dermally in the *in vivo* animal toxicity studies were converted to human equivalent doses (HED) using the body surface area (BSA) (Reagan-Shaw et al. 2008):1

In Eq. 1, *K*_*m*_ is the body weight divided by the BSA for a given species. Skin absorption kinetics depend on the total amount of chemical deposited onto the body as well as the area of deposition (Krüse and Verberk 2008). For this reason, dermal doses are expressed in units of mass per unit area of exposed skin (mass/cm^2^) (van de Sandt et al. 2007) per day. The application area for human skin penetration simulations, *A* [cm^2^], was calculated from the animal skin application area using the ratio of human to animal BSA. We used a human BSA of 1.6 m^2^, corresponding to a 60-kg adult (Reagan-Shaw et al. 2008). The final dermal human dose is:2

### Calculation of flux cleared from dermis into systemic circulation

The penetration of chemicals through skin was simulated using the transient model for *in vivo* human skin penetration developed by Kasting and co-workers (described in (Dancik et al. 2013)). The Kasting model predicts penetration through the stratum corneum, the viable epidermis and the dermis. Effects of protein binding and volume exclusion on partition and diffusion coefficients in each skin layer are taken into account to model the concentration of unbound solute in the tissue, that is, the concentration which usually drives toxicity (Blaauboer 2010). Both the polar and the lipid pathway are included in the model. Models of skin penetration incorporating both pathways predict the permeability of hydrophilic molecules better than models of the lipid phase only (Chen et al. 2012). As a result, inclusion of the polar pathway impacts the identification of risk following dermal exposure (Kupczewska-Dobecka et al. 2010). Furthermore, the model can predict the penetration of volatile chemicals and assess the impact of occlusion vs. non-occlusion on exposure and absorption (Ngo et al. 2009). It accommodates a variety of exposure scenarios (finite and infinite single doses, multiple exposure and/or removal steps). Finite doses and multiple and repeated dose scenarios are more relevant to real-life exposure to chemicals, e.g., pesticides (van de Sandt et al. 2007; Ngo et al. 2009).

For all skin penetration simulations, a skin surface temperature of 32°C and a wind velocity of 0.17 m/s were assumed. The skin structural parameters used in the model are summarized in (Dancik et al. 2013) and (Ibrahim et al. 2012).

The Kasting model simulates penetration through the lipid and polar pathways of the skin separately. From the simulations we obtained the time-dependent flux  and cumulative amount  (i = lipid or polar pathway) of permeant cleared from the dermis into the systemic circulation. The total flux and cumulative amount of material cleared into the systemic circulation at steady-state were estimated by adding the lipid and polar pathway contributions:34

The total flux and cumulative amounts calculated according to Eqs. 3 and 4 should be understood as upper limits, as these equations do not take into account the possible transfer of solute across polar / lipid pathway boundaries. The simulated repeated-dose scenarios used in the animal studies yield fluctuations in the flux and cumulative amount profiles. For this reason we calculated the average total fluxes and cumulative amounts at steady-state (Kubota et al. 2002):56

In Eqs. 5 and 6, *τ* designates the dosing interval (24 h) and *n* is a number large enough to ensure steady-state has been reached (Kubota et al. 2002). Depending on the total simulation time for a given chemical, n = 7 to 10 was chosen for the average steady-state flux calculations (Eq. 5). For the average steady-state cumulative amounts (Eq. 6), we used the final 24 h-dosing interval in each simulation in order to report the maximum amount of chemical cleared systemically.

### Coupling of the Kasting skin penetration and PBPK models

The Kasting model was originally designed as a stand-alone model for predicting skin penetration. Its current setup assumes the permeant concentration at the lower boundary of the dermis and the plasma concentration equal to zero. In our implementation, we connect the Kasting model and the PBPK model used to estimate steady-state plasma concentration, , via the flux  (Eq. 5). Using the application area for human skin penetration simulations and assuming a weight of 60 kg, this flux is converted to a human dose [mg/kg/day]. This dose is then used as the input to the PBPK model.

### PBPK model structure and simulations

All PBPK model simulations were performed using a generic modeling approach within ADME Workbench (version 1.1.31.2; AEgis Technologies Group, Inc.). The model was comprised of 15 compartments, including adipose, bone, brain, gut, heart, kidney, liver, lung, muscle, pancreas, prostate, skin, spleen, testes and thymus. Tissue compartments were linked by venous and arterial blood compartments. Chemical distribution within each tissue was assumed to occur instantaneously and each tissue was assumed to be perfusion rate-limited. These models are considered suitable for small chemicals. Coefficients describing partitioning between the tissues and blood were calculated within ADME Workbench using a mechanistic unified algorithm developed by Peyret and co-workers (Peyret et al. 2010). Log *K*_ow_, *MW*, pKa, were predicted using Advanced Chemistry Development Labs (version 12.0) and fraction unbound (*fu*) in plasma from the ChemSilico (version 1.6.1) software. Metabolism of each compound was assumed to occur only in the liver and was described as a linear (non-saturable) process set equal to 0.1 × hepatic blood flow (e.g., 0.15 L/h/kg BW). For each scenario, PBPK model simulations were performed as a constant infusion at a rate equal to the average steady-state dermal flux calculated from Eq. 5. This simplified workflow, with negligible hepatic extraction, results in an estimate of the upper limit of the steady-state plasma concentration corresponding to the simulated dermal exposure.

### Reverse dosimetry methodology

Following Judson et al. (Judson et al. 2011), we express the exposure conversion factor (ECF) as the ratio of steady-state plasma concentration to the human dermal dose:7

### Simulated exposure scenarios and application conditions

Table 1 shows that very heterogeneous exposure scenarios and application conditions were used in the *in vivo* animal studies. We systematically varied the original study designs to quantify the impact of individual aspects of the exposure scenarios and application conditions on the ECF. Table 2 provides an overview of the full set of simulations we conducted.

**Table 2:** **Overview of simulations**

Chemical	Scenario A (animal study scenario)			Scenario B	Scenario A occluded	Scenario A non-occluded	Scenario A neat application	Scenario A 1:1 (v/v) water dilution	Scenario A 1:9 (v/v) water dilution
	Dose 1	Dose 2	Dose 3						
BR	✓	✓	✓	✓	✓	✓ ^(b)^	✓ ^(b)^	✓	✓
CAP	✓	×^(a)^	×^(a)^	✓	✓ ^(b)^	✓	✓ ^(b)^	✓	✓
DGMME	✓	✓	✓	✓	✓ ^(b)^	✓	✓ ^(b)^	✓	✓
DGMBE	✓	✓	✓	✓	✓	✓ ^(b)^	✓	Different water dilutions ^(c)^	
DMF	✓	✓	✓	✓	✓ ^(b)^	✓	✓ ^(b)^	✓	✓
2-EH	✓	✓	✓	✓	✓ ^(b)^	✓	✓ ^(b)^	✓	✓
MPA	✓	✓	×	✓	✓ ^(b)^	✓	✓ ^(b)^	✓	✓
2-ME	✓	×	×	✓	✓ ^(b)^	✓	✓ ^(b)^	✓	✓
TGA	✓	✓	✓	✓	✓	✓ ^(b)^	✓	Ethanol-water dilution ^(d)^	

Scenario A refers to the daily exposure scenario and application condition implemented in each animal study. Scenario B is identical to scenario A but, when applicable, without the daily removal step. The next four scenarios are identical to scenario A but with one change in the application conditions with respect to occlusion and the presence of a vehicle. Depending on scenario A, occlusion is replaced by non-occlusion and neat application is replaced by application in a water vehicle, or vice-versa.

×: No further doses in animal studies.

^(a)^ Different applied animal doses in mg/kg/day and areas of application [cm^2^] yield one applied dose in μg/cm^2^/day.

^(b)^ These application conditions are the same as in Scenario A (animal study scenario).

^(c)^ DGMBE was applied in a 3 mL/kg water vehicle at each dose used in Scenario A (animal study scenario).

^(d)^ TGA was applied in a 1:1 (v/v) ethanol (95%) – water vehicle in Scenario A (animal study scenario).

### Implementation of different application conditions for skin penetration

Table 3 summarizes the implementation of occlusion vs. an open system and neat application vs. the presence of a vehicle in the Kasting skin penetration model. Two chemicals, DGMBE and TGA, were applied in aqueous solutions in the *in vivo* animal studies; the others were applied neat (Table 1). We compared the ECFs of DGMBE and TGA resulting from the application condition used in the respective animal studies to the ECFs calculated from neat application. For the other chemicals, we compared the ECFs from neat application to ECFs obtained from a 50% water / 50% chemical mixture (v/v) and from a 90% water / 10% chemical mixture (v/v), following the dilutions investigated by Wang et al. (Wang et al. 2009). In these dilutions the simulated amount of each chemical applied to the skin (in μg/cm^2^) is identical to the amount applied neat.

**Table 3:** **Implementation of occlusion and presence of an aqueous vehicle in the Kasting skin penetration model**

	Neat application	Application in aqueous solvent
Lack of occlusion	- Permeant vapor pressure (Table 1) specified.	- Permeant vapor pressure (Table 1) specified.
	- Partially hydrated skin in lipid pathway part of the model. In the porous pathway part of the model, fully hydrated skin is the only option.	- For solvent dose < 100 mg/cm^2^, partially hydrated skin in lipid pathway, otherwise, fully hydrated skin.
Occlusion	- Low volatility, permeant vapor pressure is assumed equal to zero.	
	- Fully hydrated skin in lipid pathway part of the model.	

### Simulation of infinite dose kinetics

We compared the average steady-state fluxes and cumulative amounts from scenarios A and B to values resulting from infinite dose applications. For the latter a single application of an arbitrarily high dose (10^7^ μg/cm^2^) was simulated. We checked that the resulting fluxes and cumulative amounts represented infinite dose kinetics by confirming that the predicted amount of chemical in the vehicle remained constant (within 1% of the applied dose) over the duration of the simulation (Selzer et al. 2012).

## Results

### Steady-state plasma concentrations (*C*_p, ss_) and ECFs from scenario A

**Figure 1:**
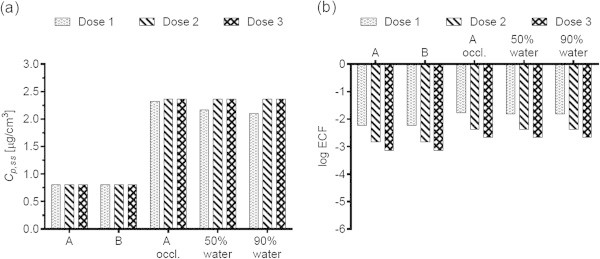
**Bayrepel (BR) (a) steady-state plasma concentrations and (b) exposure conversion factors in logarithmic scale.** Doses 1, 2, and 3 refer to animal doses listed in Table 1. Exposure scenarios A and B and modifications to scenario A are described in Table 2.

**Figure 2:**
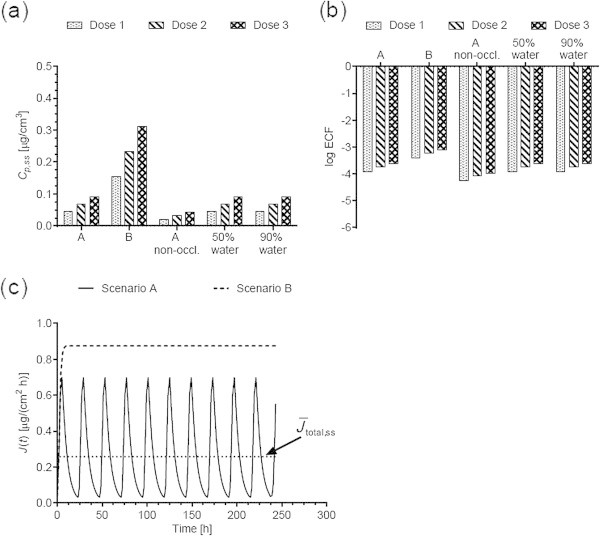
***trans*** **-Capsaicin (CAP) (a) steady-state plasma concentrations and (b) exposure conversion factors in logarithmic scale.** Dose, exposures scenarios and application conditions are described in Tables 1 and 2. (**c**) Comparison of the steady-state flux cleared from the dermis into the systemic circulation obtained from scenarios A and B. Scenario A yields a finite dose flux while B yields a steady-state flux equivalent to an infinite dose application. The solid horizontal line shows the average steady-state flux calculated (Eq. 5) calculated for scenario A.

**Figure 3:**
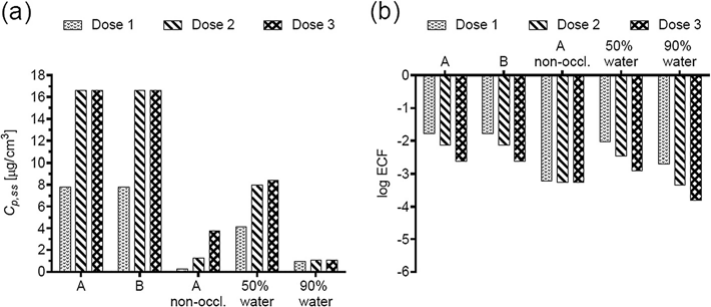
**Diethylene glycol monomethyl ether (DGMME) (a) steady-state plasma concentrations and (b) exposure conversion factors in logarithmic scale.** Doses, exposures scenarios and application conditions are described in Tables 1 and 2.

**Figure 4:**
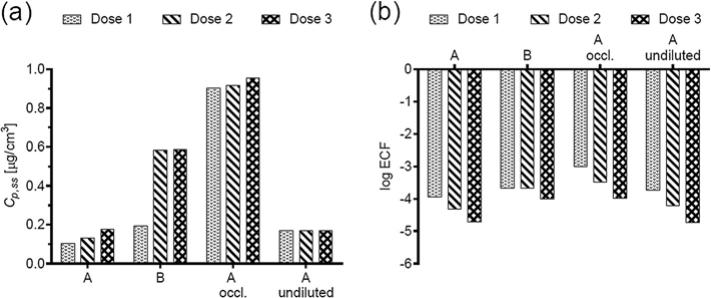
**Diethylene glycol mono-n-butyl ether (DGMBE) (a) steady-state plasma concentrations and (b) exposure conversion factors in logarithmic scale.** Doses, exposures scenarios and application conditions are described in Tables 1 and 2.

**Figure 5:**
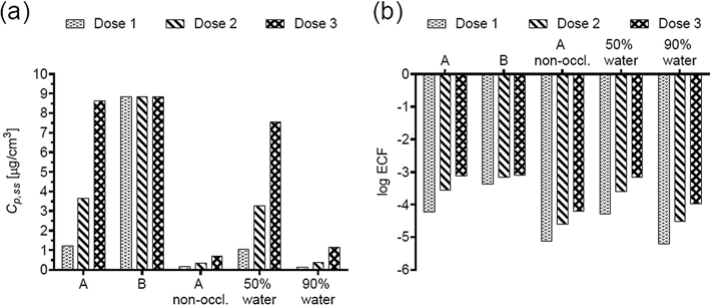
**Dimethylformamide (DMF) (a) steady-state plasma concentrations and (b) exposure conversion factors in logarithmic scale.** Doses, exposures scenarios and application conditions are described in Tables 1 and 2.

**Figure 6:**
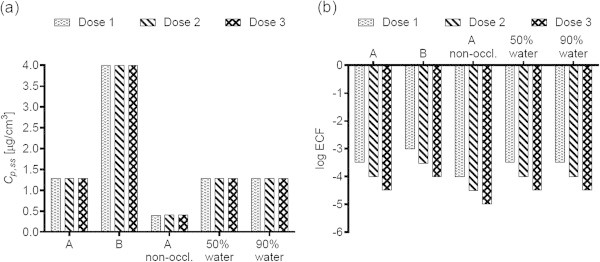
**2-Ethylhexanol (2-EH) a) steady-state plasma concentrations and (b) exposure conversion factors in logarithmic scale.** Doses, exposures scenarios and application conditions are described in Tables 1 and 2.

**Figure 7:**
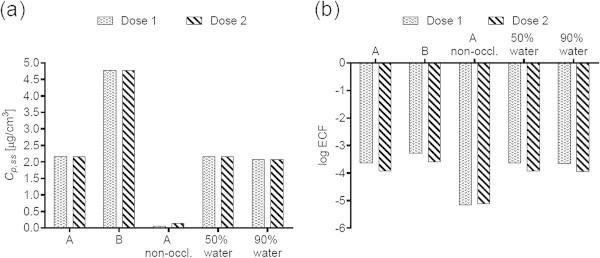
**2-Methoxypropyl-1-acetate (MPA) a) steady-state plasma concentrations and (b) exposure conversion factors in logarithmic scale.** Doses, exposures scenarios and application conditions are described in Tables 1 and 2.

Figures 1(a), 2(a), 3(a), 4(a), 5(a), 6(a), 7(a), and 9(a) show that *C*_p, ss_ either increases with the applied dose, meaning the kinetics of skin penetration are dose-dependent, or remains constant. It is constant for BR (Figure 1(a)), DGMME at the two highest doses (Figure 3(a)), 2-EH (Figure 6(a)) and MPA (Figure 7(a)). In the cases of BR and DGMME, the exposure scenario lacks a removal step. Scenario A is equivalent to scenario B and yields infinite dose kinetics at all (BR) or the highest (DGMME) doses (see comparison of scenario A and B results below). In the cases of 2-EH and MPA, infinite dose kinetics are reached after each daily application, but due to the removal step, the average steady-state flux () and *C*_p, ss_ values are less than from an infinite dose.

For all chemicals except CAP (Figure 2(b)) and DMF (Figure 5(b)), the ECF decreases with increasing dose. In the cases of CAP and DMF, the results are due to peculiarities in the animal study designs. In the case of CAP, animal doses of 64, 96 and 128 mg/kg/day were applied on application areas of 1600, 2400 and 3200 cm^2^, respectively. While this yields a constant human dermal dose of 389 μg/cm^2^/day for the skin penetration model, the corresponding PBPK model inputs are 0.166, 0.248 and 0.331 mg/kg/day. The *C*_p, ss_ values increase with the PBPK model inputs, but correspond to one human dermal dose, yielding increasing ECFs.

In the case of DMF, doses 1, 2 and 3 in Figures 5(a) and (b) correspond to 100, 200 and 400 mg/kg/day (Table 1). The areas of application to the animals used with these doses are 704, 298 and 96 cm^2^, respectively. Converting the animal doses to human applied doses (Eqs. 1, 2) yields, respectively, 20270, 13031 and 11057 μg/cm^2^/day, the human dermal doses we used in the skin penetration simulations. It follows that while the ECF decrease with increasing human dermal doses, they increase with increasing animal doses.

### Effect of switching from scenario A to B on *C*_p, ss_ and ECFs

For all chemicals applied with a removal step in the animal studies, the *C*_p, ss_ and ECFs obtained from scenario B are either equal to those from scenario A, or larger. The greatest increase is obtained for DMF dose 3 (Figure 5).

Table 4 compares the average steady-state fluxes and cumulative amounts (Eqs. 5 and 6) calculated from simulating exposure scenarios A, B and exposure to an infinite dose. For all chemicals, scenario A yields finite dose kinetics, that is, the resulting steady-state flux is less than the flux obtained from an infinite dose. Scenario B yields fluxes and cumulative amounts equal to or within 4% of infinite dose values for 7 of the 9 chemicals, at all doses. Figure 2(c) illustrates the difference between scenario A and B fluxes for CAP. In the case of DGMBE, scenario B yields kinetics within 4% of infinite dose kinetics only for the highest applied dose, whereas all 3 TGA doses yield finite dose kinetics.

**Table 4:** **Average steady-state flux and cumulative amount from exposure scenarios A, B and an infinite dose**

Chemical	Applied human doses [μg/cm^2^]	[μg/(cm^2^ · h)]			[μg/cm^2^]		
		Scenario A	Scenario B	Infinite dose	Scenario A	Scenario B	Infinite dose
BR	361.97	/ ^(a)^	2.1	2.1	/ ^(a)^	8.0	8.0
	723.94						
	1447.88						
CAP	389.19	0.26	0.87	0.87	1.4	4.6	4.6
DGMME	456.08	/ ^(a)^	33	70	81	130	280
	2280.41	/ ^(a)^	70		86	280	
	6841.22						
DGMBE	912.16	0.43	0.82	4.0	0.66	1.3	6.9
	2736.49	0.55	2.5		0.87	4.0	
	9121.62	0.75	3.9		1.25	6.6	
DMF	11056.51	110	270	270	170	410	410
	13030.89						
	20270.27						
2-EH	3949.57	19	58	58	85	300	300
	13165.23						
	39495.63						
MPA	9213.76	9.2	20	20	14	30	30
	18427.52						
2-ME	13165.23	140	340	350	150	370	370
TGA	314.21	0.33	0.67	8.9	0.35	0.95	23
	785.53	0.82	1.68		0.88	2.4	
	2041.39	2.1	4.33		2.3	6.2	

(^a^) Only scenario B in animal study.

### Effect of occlusion vs. lack thereof on *C*_p, ss_ and ECFs

For all chemicals, occlusion increases the *C*_p, ss_ and ECFs, up to 40-fold for DGMME (Figure 3(a), (b)). In general the factor increase diminishes with increasing applied dose, only for BR and CAP is it independent of dose.

### Effect of changing the vehicle on *C*_p, ss_ and ECFs

**Figure 8:**
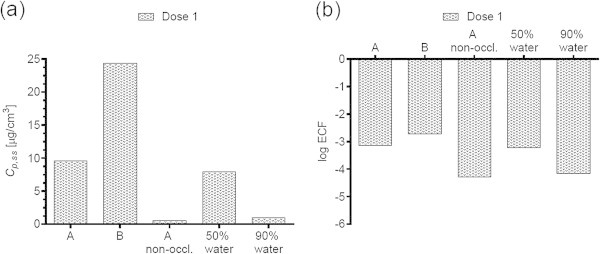
**2-Methoxyethanol (2-ME) a) steady-state plasma concentrations and (b) exposure conversion factors in logarithmic scale.** Dose, exposures scenarios and application conditions are described in Tables 1 and 2.

**Figure 9:**
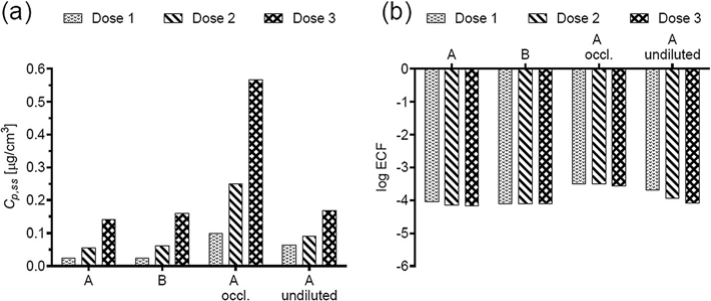
**Thioglycolic acid (TGA) steady-state plasma concentrations and (b) exposure conversion factors in logarithmic scale.** Doses, exposures scenarios and application conditions are described in Tables 1 and 2.

The effect of adding or removing an aqueous vehicle on the ECF varies with the chemical and the dilution. Addition of 50% water alters the *C*_p, ss_ and ECFs significantly only for BR (Figure 1(a), (b)) with a 3-fold increase in the values across the doses, and for DGMME (Figure 3 (a), (b)) with a 2-fold decrease in the values. The 90% water dilution has a more diverse effect, increasing the BR *C*_p, ss_ and ECF values by the same amount as the 50% dilution, but decreasing those of DGMME, DMF (Figure 5(a), (b)) and 2-ME (Figure 8(a), (b)) 8- to 16-fold. Removal of the vehicle used in the DGMBE and TGA animal studies increases the *C*_p, ss_ and ECF values up to 3-fold (Figure 4(a), (b) and 9(a), (b)).

## Discussion

The computational framework presented in this study allows for the calculation of systemic exposure from a variety of exposure scenarios and application conditions. We showed the extent to which a change in one element of an exposure protocol, for example, applied dose, occlusion vs. non-occlusion, or the use of an aqueous vehicle vs. neat application, can alter the kinetics of skin penetration and, therefore, uptake at the site of action (the plasma or an organ). Our work not only supports the conclusions of others, namely the need to assess effects based internal dose or concentration, but we also propose a pragmatic tool to facilitate the necessary calculations.

For each of the 9 chemicals investigated, we systematically studied the impact of the applied dose and of varying the exposure scenario and application conditions on *C*_p, ss_. The largest differences in *C*_p, ss_ values were obtained from switching between occlusion and non-occlusion and water-diluted (with 90% water) vs. neat application. Occlusion increases skin penetration of many compounds due to increased hydration of the stratum corneum and, in the case of volatile compounds, prevention of evaporation (Hafeez and Maibach 2013). The presence of a large volume of water in the 90% dilution affected the *C*_p, ss_ of the most hydrophilic and water-soluble chemicals (DGMME, DMF and 2-ME) to the greatest extent. Over the course of the simulations these chemicals were to a large extent retained in the water vehicle due to the high solubility, thereby reducing the amount penetrating through the skin.

In the current implementation of the framework, the Kasting skin penetration model enforces a sink boundary condition at a depth of 2 mm (taken as the “bottom” of the dermis) and in the plasma. The resulting average steady-state flux of permeant cleared from the dermis into the systemic circulation (, Eq. 5) is used as a constant input in the PBPK model to obtain estimates of the steady-state plasma concentration *C*_p, ss_. The impact of the sink boundary conditions on *C*_p, ss_ depends on the chemical. The penetration of a permeant to a depth of 2 mm in the dermis and below, and its availability for systemic uptake, depend on its physicochemistry and, significantly, on its tissue and plasma protein binding affinity (Anissimov and Roberts 2011; Dancik et al. 2012). To our knowledge, experimental binding and capillary permeability for the chemicals investigated herein have not been published. For highly protein-binding molecules with high capillary permeability, the sink boundary conditions may underestimate the dermal concentration and the plasma concentration *C*_p, ss_. For chemicals which do not penetrate significantly into the dermis due to cutaneous metabolism or sequestration, and with low capillary permeability, the dermis and plasma sink boundary condition are realistic assumptions. Taken alone, the *C*_p, ss_ = 0 condition is also appropriate for chemicals with high total body clearance (from the liver, kidneys, and/or other organs) for a given total rate of elimination (due to hepatic metabolism, renal excretion and/or elimination from other organs). Hepatic and renal clearance depend significantly on the chemical’s lipophilicity and plasma protein binding affinity (Schmidt et al. 2010; Smith et al. 2010). The model also does not incorporate feedback from the PBPK model to the skin penetration model, that is, redistribution from the systemic system into the skin tissue, the extent of which also depends on protein binding affinity (Cross et al. 1996).

In the absence of chemical-specific elimination data, PBPK model simulations can be performed using conservative and bracketing assumptions regarding metabolism in the liver and excretion in the kidney. For simplification purposes, simulations in this study were performed assuming metabolism occurs in the liver only. Metabolic clearance was described based on the hepatic extraction ratio (ER) set to 0.1. This assumption classifies each compound as a low-extraction chemical (ER < 0.3 (Rowland and Tozer 2011)). The incorporation of chemical-specific metabolism data can be used to refine the initial approach. In the absence of experimental values, current knowledge on the physicochemical properties of the chemical and its mode of action can be used to approximate key ADME (absorption, distribution, metabolism and excretion) descriptors. Hepatic metabolism is significant for BR (Antwi et al. 2008), CAP (Reilly and Yost 2006; Chanda et al. 2008), DMF (Gescher 1993) and 2-EH (Diliberto et al. 1996) and likely significant for the glycol ethers DGMME and DGMBE. Johanson et al. have reported a ER of 0.42 for the glycol ether 2-butoxyethanol (ethylene glycol monobutyl ether) in a normal perfused rat liver (Johanson et al. 1986). For these chemicals at least, an ER of 0.1 overestimates the steady-state plasma concentrations (*C*_p, ss_), assuming a linear dependence on hepatic clearance. In addition to hepatic metabolism, cutaneous metabolism is significant at least for glycol ethers (Traynor et al. 2007), DMF (Mraz and Nohova 1992) and 2-EH (Deisinger et al. 1994) and may be of some importance for CAP (Chanda et al. 2008). Factoring the skin first-pass effect into the PBPK model may further reduce the predicted *C*_p, ss_ values. For certain chemicals, metabolites may need to be tracked in a whole-body PBPK model, as these may cause toxicity (e.g., DGMME metabolites (Scofield et al. 2006)).

The framework constructed in this study offers an opportunity to refine the methodology of risk assessment by calculating the plasma concentration in a realistic exposure scenario. Current methods rely on arbitrary default absorption values, or, when oral or respiratory toxicity data are available, on route-to-route extrapolations which are appropriate under stringent conditions only (McDougal and Boeniger 2002; Rennen et al. 2004). Clearly the risk assessor must be aware of and take into account the exposure scenario(s) and the application condition(s) under which the chemical comes into contact with the skin to determine safe dermal doses. For a given chemical, there may be as many steady-state plasma concentrations and corresponding ECF values as there are ways of being exposed to that chemical in the workplace or environment in which it is found.

This workflow can be useful in interpreting results from existing dermal *in vivo* studies. Within the set of animal studies considered, the chemicals were applied to the skin of the animals without consideration of kinetics. Using our tool it is possible to better understand the relationship between external NOAEL doses determined in these studies and internal exposure.

In addition, our results apply to any risk assessment calculation based on the kinetics of skin penetration, one example being the calculation of Margins of Safety (MoS) for topically applied chemicals (Nohynek et al. 2004; Soeborg et al. 2007). There is a potential for further refining risk assessment by connecting the workflow presented here to models predicting dermal loads of chemicals based on worker and consumer habits, such as ConsExpo or RISKOFDERM (Marquart 2010). Results of this work also have implications for read-across, which consider kinetics only via the examination of physicochemical parameters (Dimitrov and Mekenyan 2010; Wu et al. 2010).

The computational framework presented herein is equally relevant to pharmacology, specifically the development of topical drugs targeting the systemic circulation system or internal organs via the transdermal route. A PBPK framework in which different topical dosage regimens can be modelled (in analogy to exposure scenarios and conditions) and compared to each other can help establish the pharmacological profile of a potential drug and serve a useful tool for bioequivalence studies of topical compounds (Shah et al. 1998; Jones et al. 2009).

Furthermore, our PBPK framework fits well into the emerging systems pharmacology approach (Vicini and van der Graaf 2013). It can be used for “model-based thinking” in drug development, by allowing the focus to be on target or site-of-action selection and validation prior to selection of a particular lead molecule. It allows for mechanistic detail to be added at various stages of the processes incorporated into the framework. This detail can be at the level of dosage and exposure, for example repeated vs. single dose, or high volatility at the skin surface. It can also be at the level of transdermal transport and/or blood and internal organ uptake and clearance. For chemicals which are highly metabolized in the skin, liver, and/or other organs, experimental data on the extent of metabolism can be easily incorporated into the workflow.

### Outlook

For a more accurate prediction of the plasma concentration profile, a seamless integration of the Kasting skin penetration model and the PBPK model is needed. This can be achieved by:

- replacing the sink boundary conditions at the bottom of the dermis and in the plasma by a boundary condition relating the dermal tissue concentration to the non-zero plasma concentration via the flux through the capillary endothelia (see Eq. 12 in (Ibrahim et al. 2012)).
- incorporating the PBPK ordinary differential equations (ODEs) into the skin penetration model ODE matrix and solving the entire system simultaneously at each time step.

The second modification would yield transient profiles of the plasma concentration. For repeated application scenarios, the transient profiles help evaluate the time to reach steady-state and the number of doses required to reach a given fraction of the steady-state concentration.

Work to improve the Kasting skin penetration model is also underway. The aim is to more accurately describe protein binding processes occurring in the skin, specifically, to incorporate reversible binding (Frasch et al. 2011; Nitsche and Frasch 2011). This will lead to more reliable predictions of toxicity, as it is the unbound concentration of a chemical which drives its kinetics and resulting toxicity (Blaauboer 2010; Yoon et al. 2012).

## Conclusion

The workflow linking Kasting’s models of skin penetration and whole-body PBPK enables estimation of plasma concentrations for various applied doses, exposure scenarios and application conditions. Through examination of real dermal *in vivo* studies, we provide examples illustrating the need to use internal steady-state plasma concentration to reduce uncertainty in risk assessment following dermal exposure.

## Abbreviations

BR: Bayrepel
CAP: *trans*-Capsaicin
DGMME: Diethylene glycol monomethyl ether
DGMBE: Diethylene glycol mono-n-butyl ether
DMF: Dimethylformamide
ECF: Exposure conversion factor
2-EH: 2-Ethylhexanol
MPA: 2-Methoxypropyl-1-acetate
NOAEL: No-observed-adverse-effect level
NOEL: No-observed-effect level
MoS: Margin of safety
2-ME: 2-Methoxyethanol
ODE: Ordinary differential equation
PBPK: Physiologically-based pharmacokinetic
TGA: Thioglycolic acid.
